# Supplementary material for: Hydrolyzable Additive-Based Silicone Elastomers: A New Approach for Antifouling Coatings
Source: Polymers (Basel). 2019 Feb 12;11(2):305. doi: 10.3390/polym11020305 (PMC6419558; doi:10.3390/polym11020305)
Supplement: Supplementary file 1 [file polymers-11-00305-s001.pdf]

polymers

Supplementary Materials

Hydrolyzable Additive-Based Silicone Elastomers: A New Approach for Antifouling Coatings

Laure Gevaux [1,*], Marlène Lejars [1], André Margaillan [1], Jean-François Briand [1], Robert Bunet [2] and Christine Bressy [1]

1 Laboratoire Matériaux Polymères Interfaces Environnement Marin (MAPIEM), Université de Toulon,EA 4323, 83957 La Garde, France; marlene.lejars@univ-tln.fr (M.L.); andre.margaillan@univ-tln.fr (A.M.); jean-francois.briand@univ-tln.fr (J.-F.B.); christine.bressy@univ-tln.fr (C.B.)

2 Institut Océanographique Paul Ricard, Ile des Embiez, 83140 Six-Fours-les-Plages, France; robert.bunet@institut-paul-ricard.org

* Correspondence: laure.gevaux@univ-tln.fr

Received: 23 January 2019; Accepted: 8 February 2019; Published: 12 February 2019

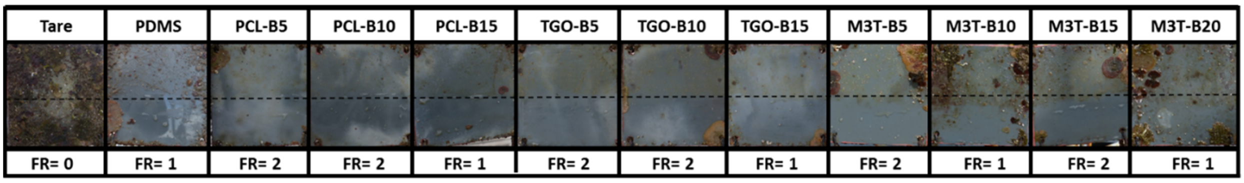

Figure S1. Evaluation of the fouling release ability after 8 months thanks to the cleaning of the lower half of the panel by means of a wetted sponge.

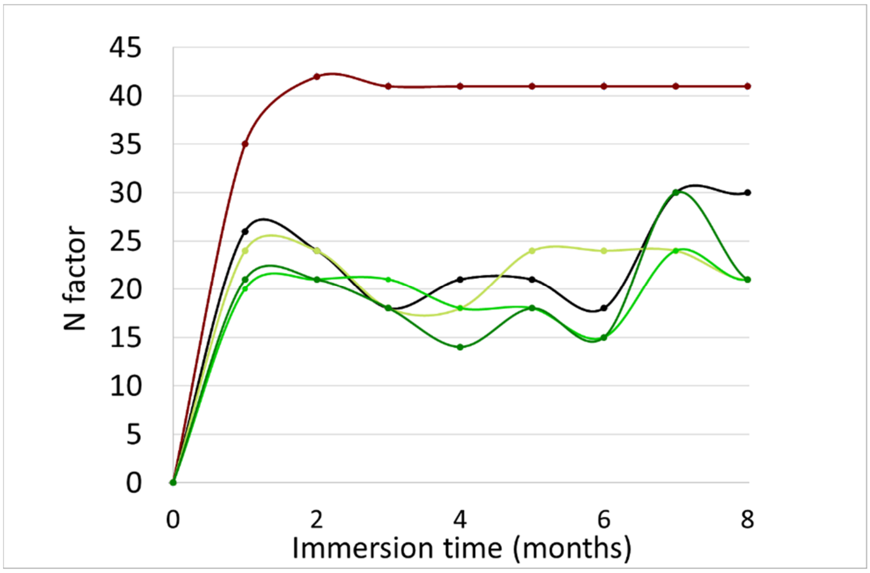

Figure S2. Evolution of the N factor of PCL-BX coated panels immersed for 8 months in Toulon bay with PVC panel (brown curve), PDMS reference (black curve), PCL-B5 (light green), PCL-B10 (medium green), PCL-B15 (dark green).

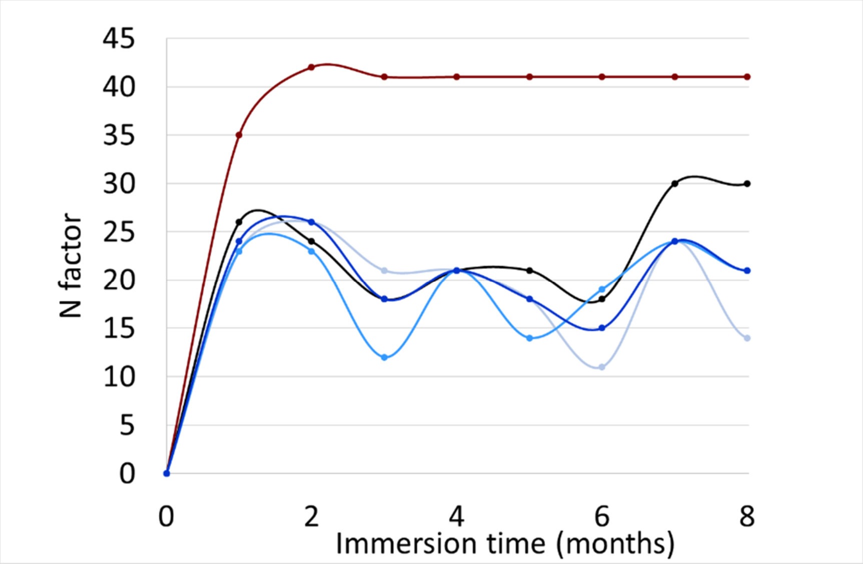

Figure S3. Evolution of the N factor of TGO-BX coated panels immersed for 8 months in Toulon bay with PVC panel (brown curve), PDMS reference (black curve), TGO-B5 (light blue), TGO-B10 (medium blue), TGO-B15 (dark blue).

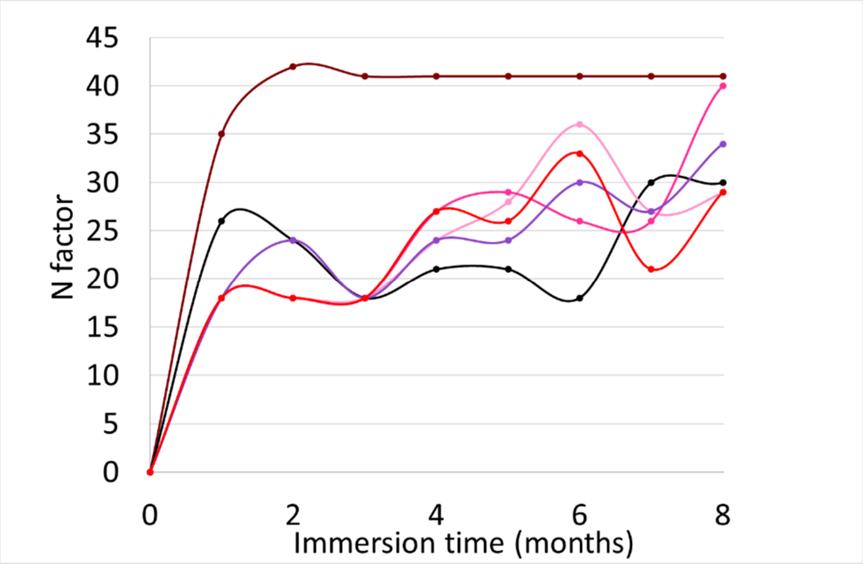

Figure S4. Evolution of the N factor of M3T-BX coated panels immersed for 8 months in Toulon bay with PVC panel (brown curve), PDMS reference (black curve), M3T-B5 (light pink), M3T-B10 (dark pink), M3T-B15 (purple) and M3T-B20 (red).

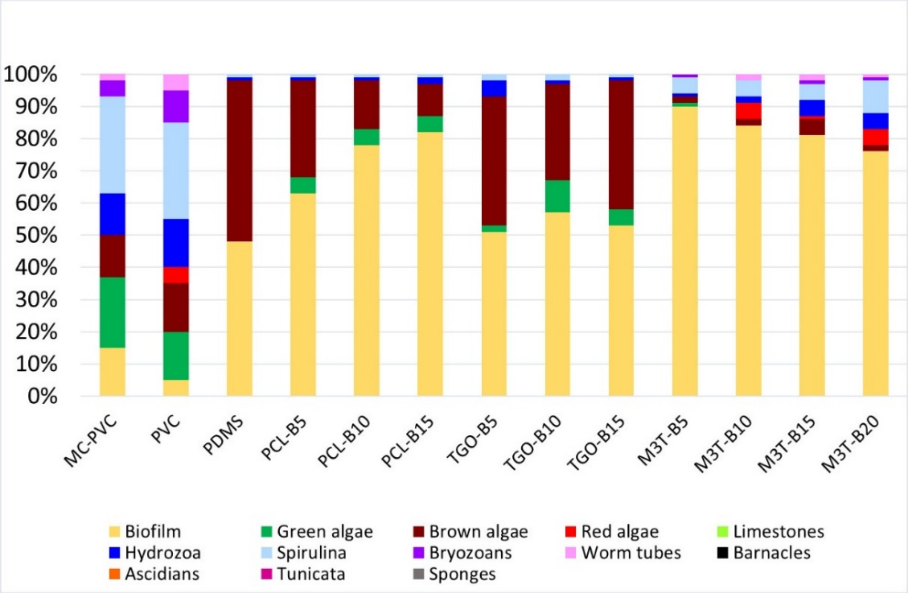

Figure S5. Determination of the percentage of the coated surface covered by several type of marine organisms for 2 months of immersion (MC-PVC corresponds to a monthly changed PVC panel to show the seasonal variations).

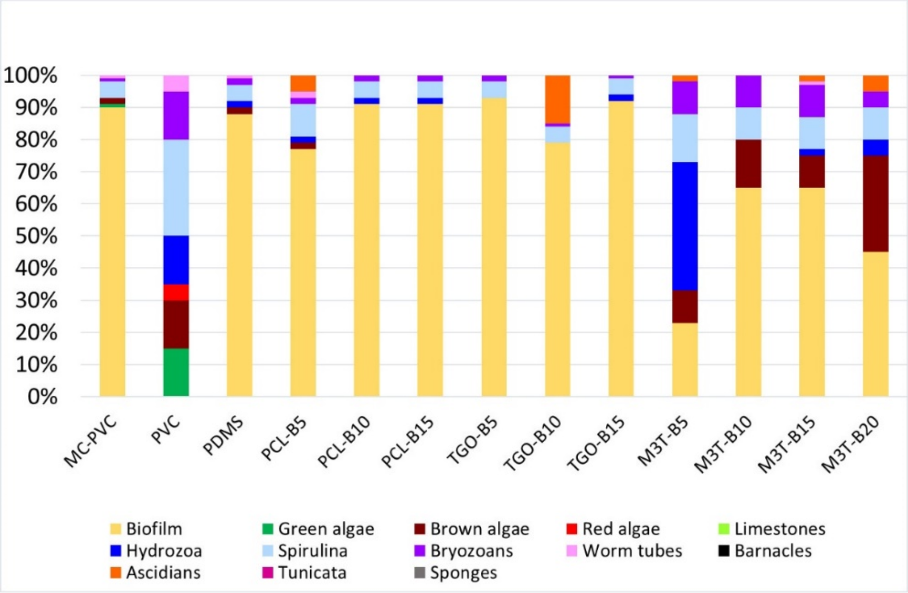

Figure S6. Determination of the percentage of the coated surface covered by several type of marine organisms for 6 months of immersion in Toulon bay (MC-PVC corresponds to a monthly changed PVC panel to show the seasonal variations).

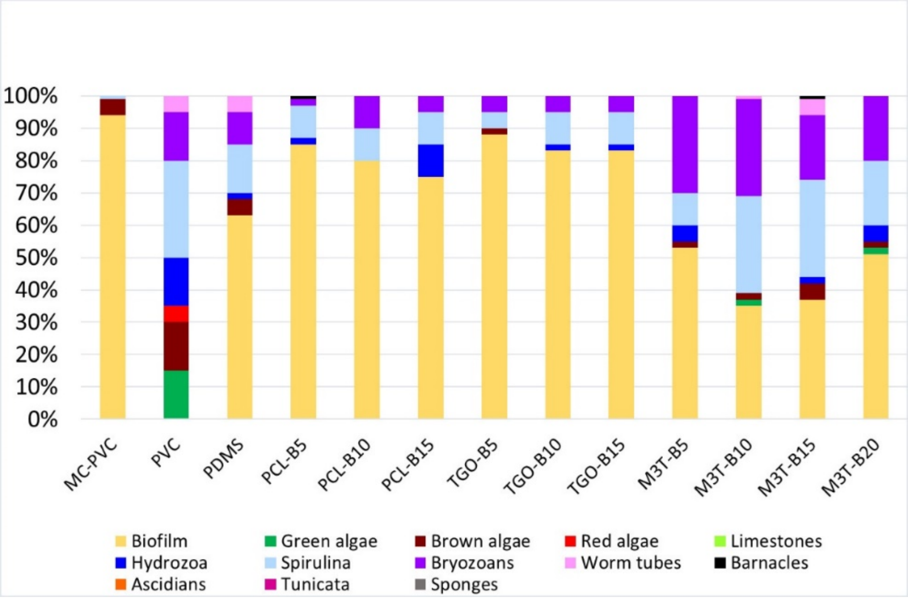

Figure S7. Determination of the percentage of the coated surface covered by several type of marine organisms for 8 months of immersion in Toulon bay (MC-PVC corresponds to a monthly changed PVC panel to show the seasonal variations).

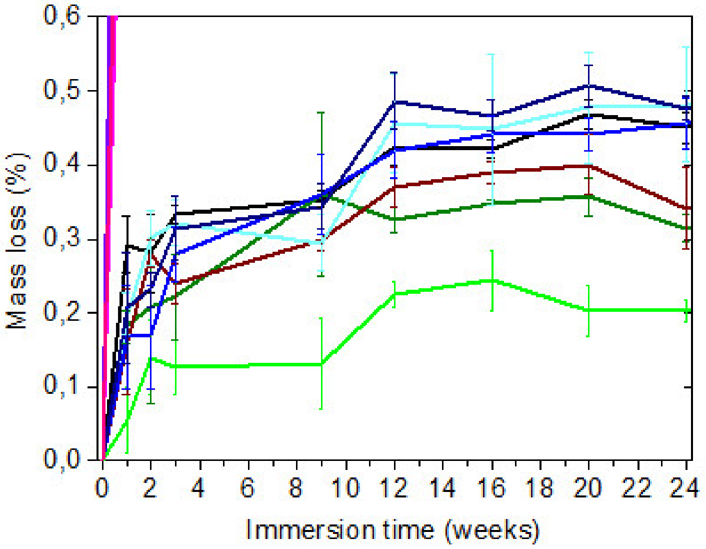

Figure S8. Mass loss (%) in deionized water at room temperature of PDMS reference (black curve), TGO-B5 (light blue), TGO-B10 (medium blue), TGO-B15 (dark blue), PCL-B5 (light green), PCL-B10 (medium green) and PCL-B15 (brown).

Table S1. Amount of cypris larvae tested on each coating, amount of fixed cypris larvae and the resulting adhesion percentage values.

Tested coating	Total tested cypris larvae	Fixed cypris larvae	Adhesion percentage (%)	Average adhesion percentage (%)	Standard deviation (%)
PS	9	6	66,7	87	16
	9	9	100,0		
	11	11	100,0		
	21	17	81,0		
PDMS	8	5	62,5	72	21
	15	15	100,0		
	6	3	50,0		
	4	3	75,0		
PCL-B5	6	0	0,0	38	35
	3	2	66,7		
	19	13	68,4		
	6	1	16,7		
PCL-B10	10	10	100,0	48	49
	8	1	12,5		
	7	0	0,0		
	10	8	80,0		
PCL-B15	7	4	57,1	25	30
	7	3	42,9		
	3	0	0,0		
	6	0	0,0		
TGO-B5	8	4	50,0	68	15
	9	7	77,8		
	27	22	81,5		
	8	5	62,5		
TGO-B10	17	15	88,2	88	9
	20	17	85,0		
	15	15	100,0		
	14	11	78,6		
TGO-B15	7	0	0,0	20	24
	7	2	28,6		
	5	0	0,0		
	10	5	50,0		
M3T-B5	5	3	60,0	79	16
	9	7	77,8		
	5	5	100,0		
	9	7	77,8		
M3T-B10	4	2	50,0	30	18
	8	1	12,5		
	5	2	40,0		
	11	2	18,2		
M3T-B15	5	0	0,0	13	19
	8	1	12,5		
	22	0	0,0		
	5	2	40,0		

M3T-B20	6	0	0,0	7	8
	9	0	0,0		
	14	2	14,3		
	15	2	13,3		

© 2019 by the authors. Submitted for possible open access publication under the terms and conditions of the Creative Commons Attribution (CC BY) license (http://creativecommons.org/licenses/by/4.0/).
